# Coffee tree architecture and its interactions with microclimates drive the dynamics of coffee berry disease in coffee trees

**DOI:** 10.1038/s41598-019-38775-5

**Published:** 2019-02-22

**Authors:** Natacha Motisi, Fabienne Ribeyre, Sylvain Poggi

**Affiliations:** 1 CIRAD, UPR Bioagresseurs, F-34398 Montpellier, France; 2 Bioagresseurs, Univ Montpellier, CIRAD, Montpellier, France; 3 IGEPP, INRA, AgroCampus Ouest, Université Rennes 1, F-35653 Le Rheu, France

## Abstract

Coffee berry disease (CBD), which is widespread in Africa, has been responsible for massive yield losses of *Coffea arabica*. In Cameroon, *C. arabica* is mainly planted in agroforestry systems on smallholder farms, where low incomes hinder the use of chemicals to manage the disease. Novel agroecological strategies for controlling CBD are expected to be implemented and even increase in the current context of global changes. In this study, we showed that coffee tree architecture and its interactions with microclimates were important to CBD cluster symptom appearance (CSA), with notable CSA increasing along the tree branch away from the trunk to the tip of the branch. As shade trees can modify microclimates, we further investigated scenarios of various microclimatic conditions under shade to explore the effects of agroforestry systems on CBD dynamics in coffee trees. We showed that shade could result in contrasting effects on disease dynamics, decreasing CSA along the branch and increasing epidemic duration. We suggest that the contrasting effects of shade on disease dynamics need further evaluation of the possible trade-offs among the variables at play, and we recommend a combination of epidemiological and architectural modelling to help design more cost-effective and environmentally friendly CBD management strategies.

## Introduction

Coffee berry disease (CBD), caused by the fungal pathogen *Colletotrichum kahawae*, is widespread in Africa and, depending on climatic conditions, has been responsible for massive coffee yield losses, with up to 90% loss recorded^1^. In many African countries, *C. arabica* is mainly cropped on smallholder farms, where low incomes hinder the use of chemicals to manage the disease. Novel agroecological strategies for controlling CBD are expected to be implemented and even increase in number due to the current context of global changes.

Coffee tree architecture may be a possible lever for managing CBD. Mouen *et al*. suggest that coffee tree architecture, in terms of branch and leaf arrangement on a tree, can influence the distribution of CBD on a tree. In fact, the authors found homogeneous disease incidence in trees showing umbrella architecture (where almost all the branches of a tree are in the same stories, preferentially the top of the tree), while they found an ascending gradient of disease incidence from the top to the base of coffee trees showing pyramidal architecture (where the branches and the stories are well differentiated from the top of a tree to its base)^2^. As *C. kahawae* mainly disperses by rain splash^3,4^, Mouen *et al*. suggest that conidia transported by gravity and raindrops would be intercepted by the branches in the canopy. However, Calonnec *et al*., who studied powdery mildew on grapevines^5^, argue that architecture is an important variable affecting disease spread in terms of “availability” of susceptible hosts (number, location and susceptibility of hosts). In the case of coffee trees, Vaast *et al*. show that pruning coffee berries increases berry maturation rate^6^. Nonetheless, susceptibility to CBD is assumed to be negatively and nonlinearly linked to berry maturation rate^2,4,7–9^. Consequently, manipulating berry distribution within the tree architecture, hereafter referred to as the arrangement of berry clusters (ensembles of berries borne by a branch node), may affect the “availability” of susceptible hosts by altering (i) distances (locations) between infected and susceptible berries, (ii) the fruit load (number) of susceptible berries, and potentially (iii) the period of berry susceptibility by modifying the maturation rate. Tree architecture is thus likely to influence the movement of inoculum within the tree, potentially providing control of the disease.

Modifications to microclimates to hinder *C. kahawae* spread and infection are another possible lever for managing CBD. Nonetheless, knowledge on the relationships between the epidemiology of CBD and microclimates is scarce. The primary inoculum is supposed to remain dormant during the dry season in flower buds, branch bark or mummified berries left on the branch^7,10^; however, the dormant form (either spores or mycelium) has not formally been identified or localized within a tree^9^. Primary infections occur at the beginning of fruit expansion (i.e., four to ten weeks after flowering), supposedly favoured by conditions of relatively low temperatures (15–25 °C) and high relative humidity (>98%) that are prevalent during the rainy season^3,9,11^. The latent period (time between host infection and appearance of symptoms) is highly variable, ranging from six days (recorded under optimal controlled conditions)^12,13^ to four weeks (recorded under outdoor field conditions)^8^. The secondary inoculum, conidia formed on the berry surface, is dispersed by rain splash, which is the main dispersal mode of *C. kahawae* on a tree (autoinfection), as well as between trees (alloinfection)^3,4^. Intensification of disease, caused by secondary infections, occurs during the green berry stage (between approximately 7 and 18 weeks after flowering), which is supposed to be the stage of highest susceptibility to CBD^2,4,7–9^.

In Cameroon, coffee trees are mainly cropped under shade trees, providing conditions similar to those of their original habitat. However, shade affects microclimates by buffering temperature^14^ and likely increasing wetness^15^, which could be favourable to *C. kahawae* fruit penetration and infection. In addition, Vaast *et al*.^6^ showed that a rather dense shade level of 45% delays coffee berry ripening by as much as one month, which could increase the period of berry susceptibility to *C. kahawae*. At the same time, numerous authors argue that agroforestry systems have the potential to control CBD^4,16–20^, notably because shade trees provide protection for coffee trees against rainfall, thereby decreasing pathogen dispersal via splashing^4,19^. Thus, the usefulness of growing coffee trees in agroforestry systems to control CBD is not clear. In particular, if shade can modify CBD propagation through the modification of microclimates, then more information is needed to understand the possible trade-offs between the effects of rainfall and microclimates under shade.

**Figure 1:**
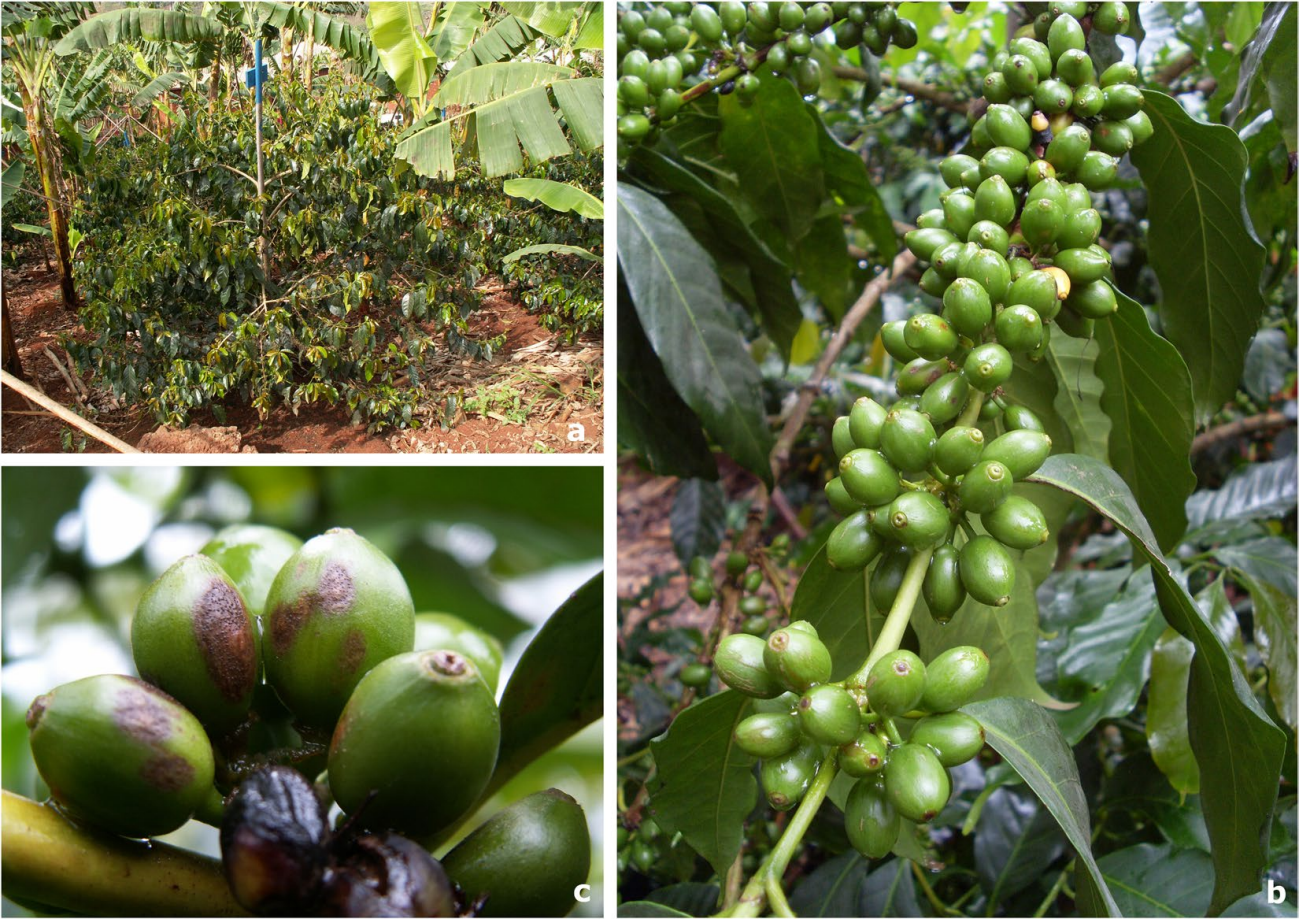
Coffee trees and coffee berry disease characteristics. (**a**) A coffee tree showing an intermediate pyramidal architecture (where branches and stories are well differentiated from the top of a tree to its base), with an umbrella architecture (where branches are almost in the same stories in a tree). (**b**) Berry clusters distributed along a branch. (**c**) Symptoms of coffee berry disease caused by *Colletotrichum kahawae*. All photographs were taken by the author Natacha Motisi.

**Figure 2:**
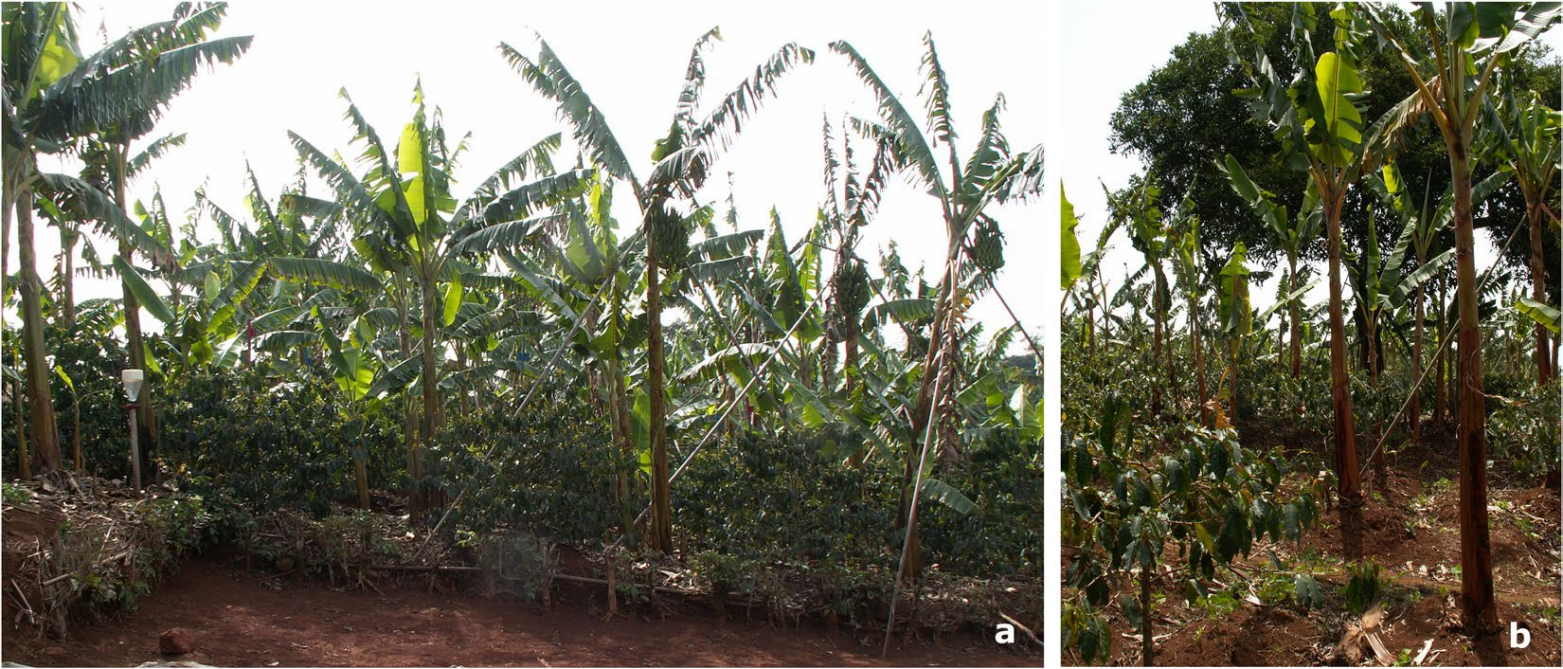
Banana shading system vs. kola shading system. (**a**) The coffee growing area in association with plantain banana trees where original data field were collected (banana shading system). (**b**) The forestry kola tree where the microclimatic data were collected over two years to investigate scenarios of modifications of coffee berry disease dynamics under agroforestry systems. All photographs were taken by the author Natacha Motisi.

**Figure 3:**
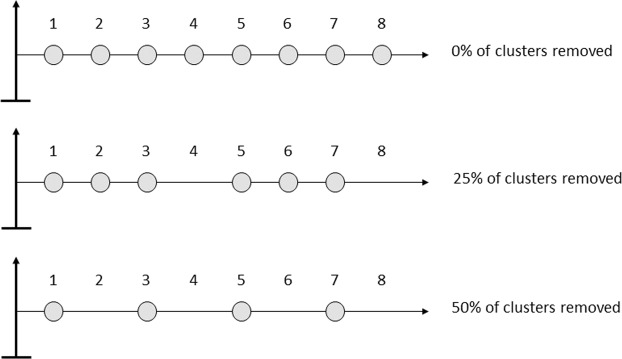
Schematic description of cluster position (with an example of 8 clusters numbered 1 to 8 along the branch) and cluster arrangement with 0% (control trees), 25% and 50% of the clusters removed along the branch.

In this study, we investigated whether coffee tree architecture can influence CBD cluster symptom appearance (CSA, Fig. 1c) and analysed tree architecture effects relative to those of microclimates. For this analysis, we carried out a field experiment on a smallholder coffee farm in Cameroon over two years (2012 and 2013) on coffee trees cropped under the partial and non-permanent shade of banana trees, hereafter referred to as the banana shading system (Fig. 2a). In terms of the effect of tree architecture on CBD, we considered various covariates (Table 1): the branch story within a tree (top, middle or lower story of a coffee tree, Fig. 1a), the spatial configuration of berry clusters (Fig. 1b) resulting from prior manual pruning of clusters along the branch (cluster arrangement, Fig. 3), the position of each cluster on a branch starting from the trunk moving to the tip of the branch, the fruit load per cluster, and berry physiological age, which we assumed to be an indicator of berry susceptibility. We monitored a set of microclimatic variables, namely, precipitation, temperature and relative humidity, to define the microclimate covariates. Precipitation, defined as cumulative rainfall, was assumed to mediate spore dispersal. Suitable conditions for infection (SCI) were computed as the number of hours beyond 5 hours of suitable temperature (15–25 °C) and relative humidity (>98%). The CBD latent period can vary between one week and one month; thus, both precipitation and suitable conditions for infection were considered within three time windows, namely, 7 to 14 days (time window 1, W1), 15 to 21 days (time window 2, W2) and 22 to 29 days (time window 3, W3) before CSA. We applied a statistical method from the field of machine learning, boosted regression trees (BRT), which enables researchers to address questions relative to complex biological systems involving many variables and characterized by multiple interactions between processes. We used this approach to assess the relationship between CSA and the previously mentioned explanatory variables by fitting a model to the field data containing disease dynamics on coffee trees cropped in the banana shading system. We subsequently studied whether agroforestry systems can modify disease dynamics by investigating *in silico* the hypothesis that the microclimate induced by forestry tree can decrease CBD levels. For this purpose, we applied a new set of microclimatic variables (precipitation, temperature and relative humidity) measured over two years under a kola shade tree (Fig. 2b) present on the coffee farm, hereafter referred to as the kola shading system, to the previous fitted model used as the reference situation. Our results provide information on (i) coffee tree architecture management as part of agroecological strategies for controlling CBD and (ii) the manner in which agroforestry systems can affect CBD dynamics at the tree scale. We encourage the use of mechanistic modelling to help design novel, more cost-effective and environmentally friendly management strategies at both the tree scale and plot scale.

**Table 1:** Names and descriptions of the variables used for boosted regression trees (BRT) analyses.

Short name	Description	Type
CSA	Cluster symptom appearance (response variable)	Qualitative (binary)
Year	2-level factor: 2012, 2013	Qualitative
*Architectural covariates*		
Branch story	Branch position in the coffee tree: top, middle, lower stories	Qualitative
Cluster arrangement	Spatial configuration of berry clusters resulting from the removal of clusters along the branch, 0, 25 and 50% removal (Fig. 3)	Qualitative
Cluster position	Position of each cluster along the branch from the trunk (position 1) to the last possible position on the branch	Qualitative
Cluster fruit load	Number of berries per cluster	Quantitative
Physiological age	Sum of temperatures (°C days) from flowering assumed to be linked to berry susceptibility to coffee berry disease	Quantitative
*Microclimate covariates*		
Precipitation W1	Cumulative rainfall in time window 1 (W1), i.e., from 7 to 14 days before the observation of CSA	Quantitative
Precipitation W2	Cumulative rainfall in time window 2 (W2), i.e., from 15 to 21 days before the observation of CSA	Quantitative
Precipitation W3	Cumulative rainfall in time window 3 (W3), i.e., from 22 to 29 days before the observation of CSA	Quantitative
SCI W1	Number of hours beyond 5 hours of suitable conditions for infection (temperatures ranging from 15 to 25 °C and relative humidity > 98%) in time window 1 (W1), i.e., from 7 to 14 days before the observation of CSA	Quantitative
SCI W2	Number of hours beyond 5 hours of suitable conditions for infection (temperatures ranging from 15 to 25 °C and relative humidity > 98%) in time window 2 (W2), i.e., from 15 to 21 days before the observation of CSA	Quantitative
SCI W3	Number of hours beyond 5 hours of suitable conditions for infection (temperatures ranging from 15 to 25 °C and relative humidity > 98%) in time window 3 (W3), i.e., from 22 to 29 days before the observation of CSA	Quantitative

## Results

### Field data

**Figure 4:**
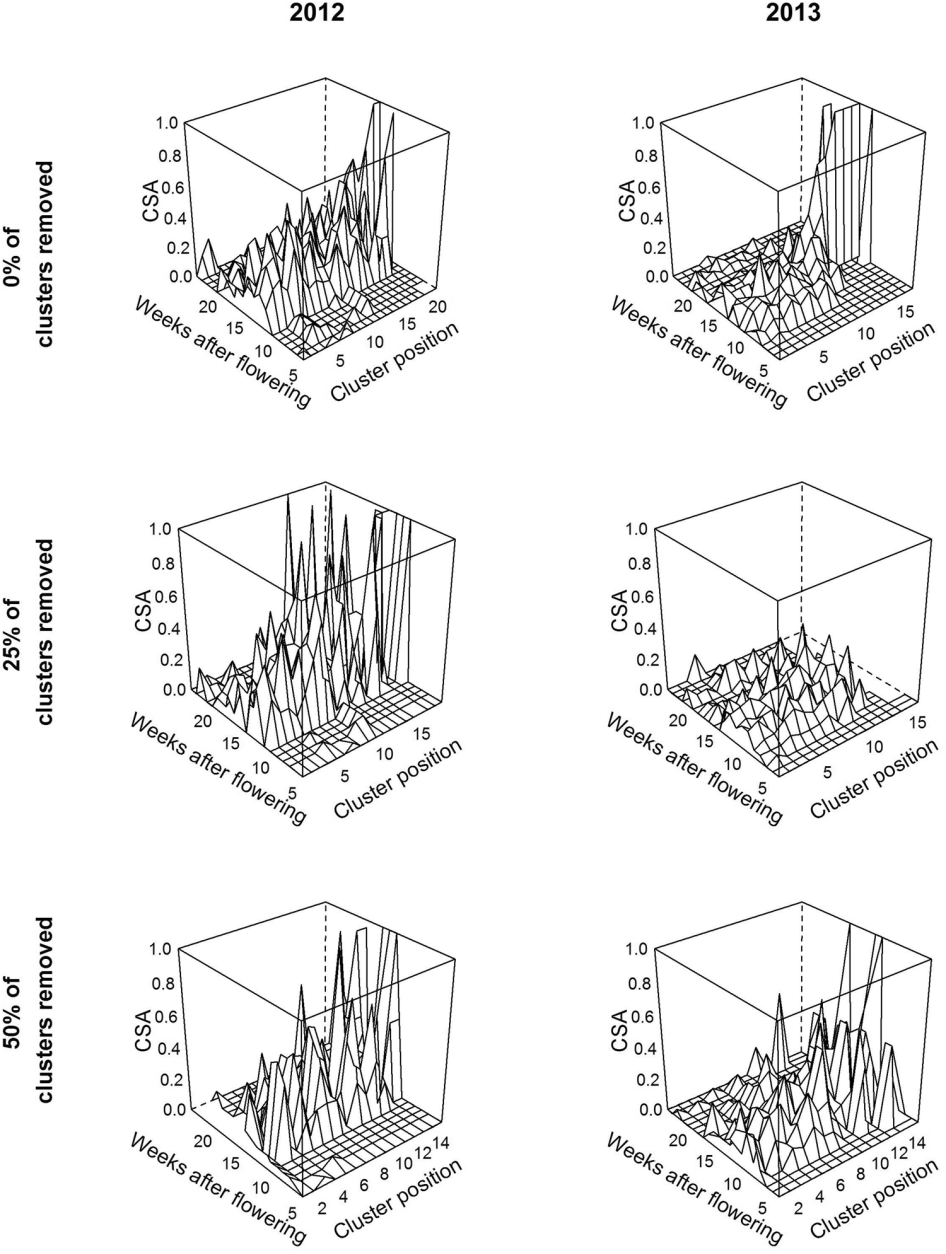
Field data showing the weekly dynamics of cluster symptom appearance (CSA) along the branch, moving away from the trunk (cluster position 1) to the tip of the branch. The rows are separated by the modalities of cluster arrangement, with 0, 25 and 50% of the clusters removed, and the columns are separated by the two years studied, 2012 and 2013.

The field data contained disease dynamics on coffee trees cropped under the shade of banana trees. Disease dispersal was investigated over three different cluster arrangements obtained by manually eliminating 0, 25 and 50% of the clusters along all the branches of the coffee trees (Fig. 3). The patterns of CSA were similar over the two years and among the three categories of cluster removal rates, except for 25% removal in 2013 (Fig. 4). In fact, regardless of the year and cluster removal rate, CSA intensified between 10 and 20 weeks after flowering, and within this period, CSA increased moving away from the trunk along the branch to its tip.

### Relative influences and effects of the explanatory variables

Using a gradient boosting regression, we fitted a model with good prediction performance (Annex 1), demonstrating the substantial role of tree architecture in symptom appearance and a strong interaction with microclimate.

**Figure 5:**
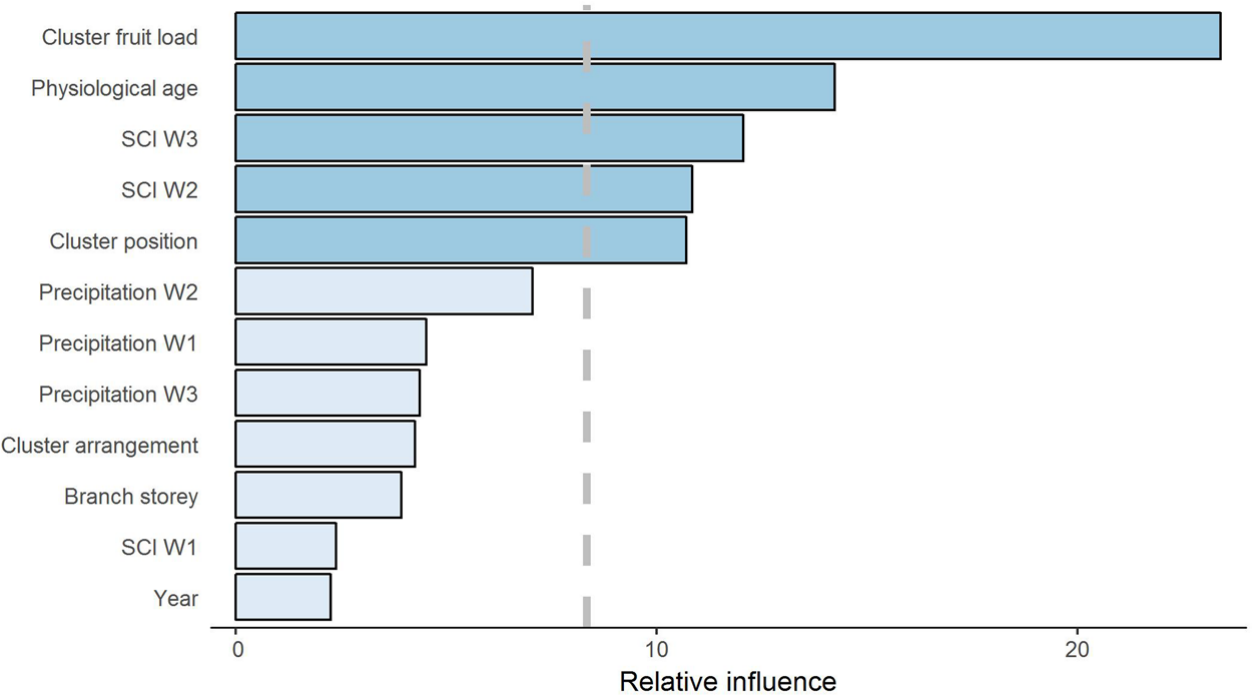
Relative influence of the predictor variables on cluster symptom appearance (CSA). The most important variables influencing CSA (dark blue) show higher relative influence than that expected due to chance (i.e., smaller than 8.3%, dashed vertical grey line). Details about the variables are provided in Table 1. SCI are the suitable conditions for infection in terms of temperature and relative humidity in the time windows W1 (7 to 14 days before the observation of CSA), W2 (15 to 21 days before CSA) and W3 (22 to 29 days before CSA).

The most important variables influencing CSA were (i) architectural covariates, namely, cluster fruit load, berry physiological age, and cluster position along the branch, and (ii) microclimate covariates, namely, the suitable conditions for infection (SCI) in terms of temperature and relative humidity in two time windows, SCI W3 (22 to 29 days before CSA) and SCI W2 (15 to 21 days before CSA) (Fig. 5).

**Figure 6:**
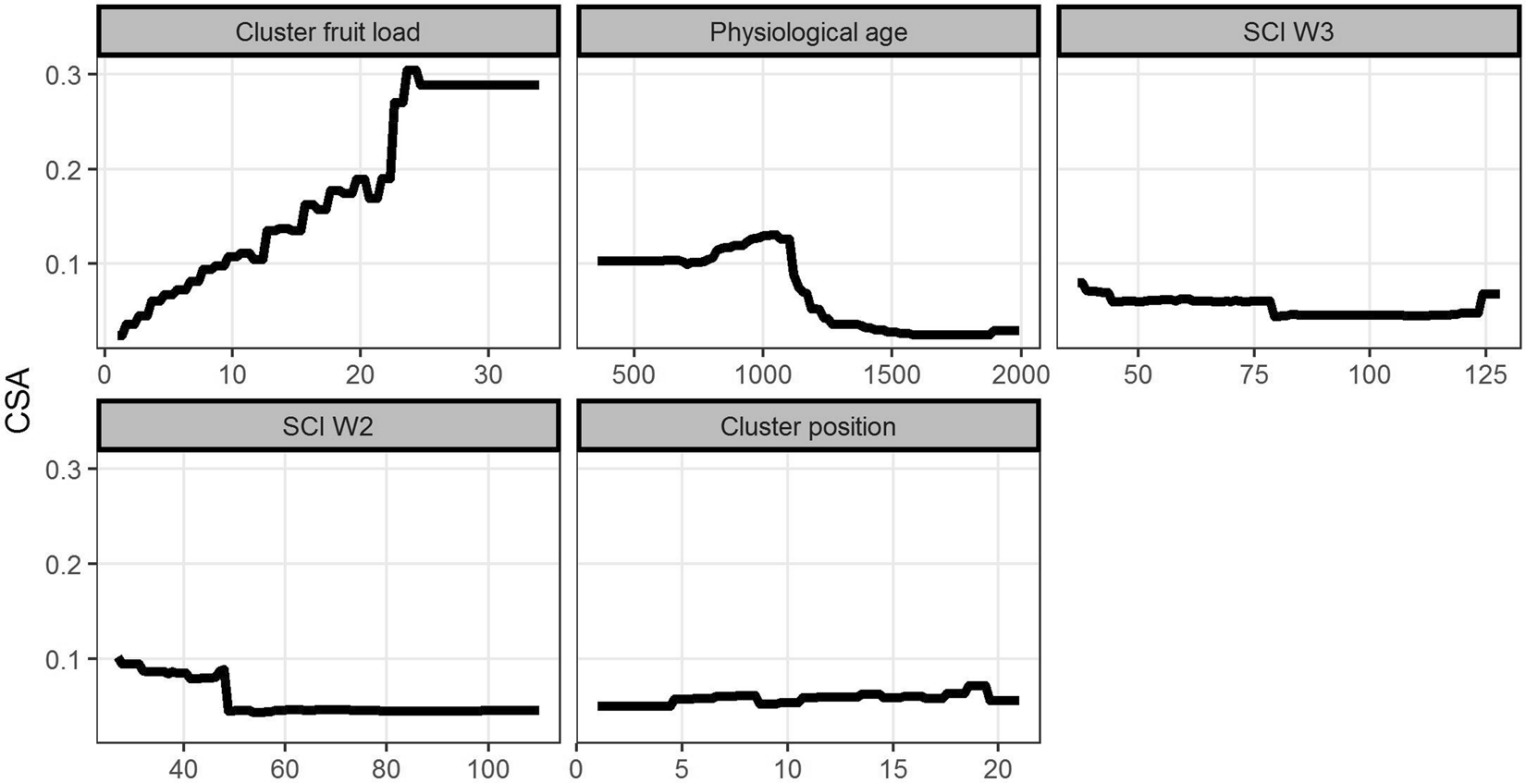
Dependence plots showing the relationships between cluster symptom appearance (CSA) and the most important variables influencing CSA (effect of the other variables fixed to their mean). The variables are described in Table 1. SCI W2 and SCI W3 are the suitable conditions for infection in terms of temperature and relative humidity in the time windows W2 (15 to 21 days before the observation of CSA) and W3 (22 to 29 days before CSA), respectively.

Cluster fruit load was the strongest predictor of CSA, with a relative importance of 23.4%, and was characterized by a positive relationship with CSA, which reached a plateau of 25 berries per cluster (Fig. 6). Berry physiological age accounted for 14.2% of the total effect on CSA and was nonlinearly and negatively related to CSA. Microclimate, through SCI W3 and SCI W2, contributed 12.1% and 10.8%, respectively, to CSA, with a counterintuitive decreasing relationship with CSA followed by a plateau for both time windows. Cluster position along the branch was the fifth most influential predictor (relative contribution of 10.7%), characterized by an increase in CSA along the branch moving away from the trunk.

The other variables showed smaller than expected influences due to chance (i.e., smaller than 8.3%, Fig. 5), namely, precipitation in time windows W1, W2 and W3; cluster arrangement along the branch (0%, 25% and 50% of clusters removal); branch story in the tree (top, middle and lower story); and SCI in W1 and year.

Interestingly, SCI W3 and cluster position were involved in interactions. The most important interaction involved SCI W3 and berry physiological age (interaction strength 0.26), and the second most important involved cluster position and SCI W1 (interaction strength 0.25).

### *In silico* experiments

**Figure 7:**
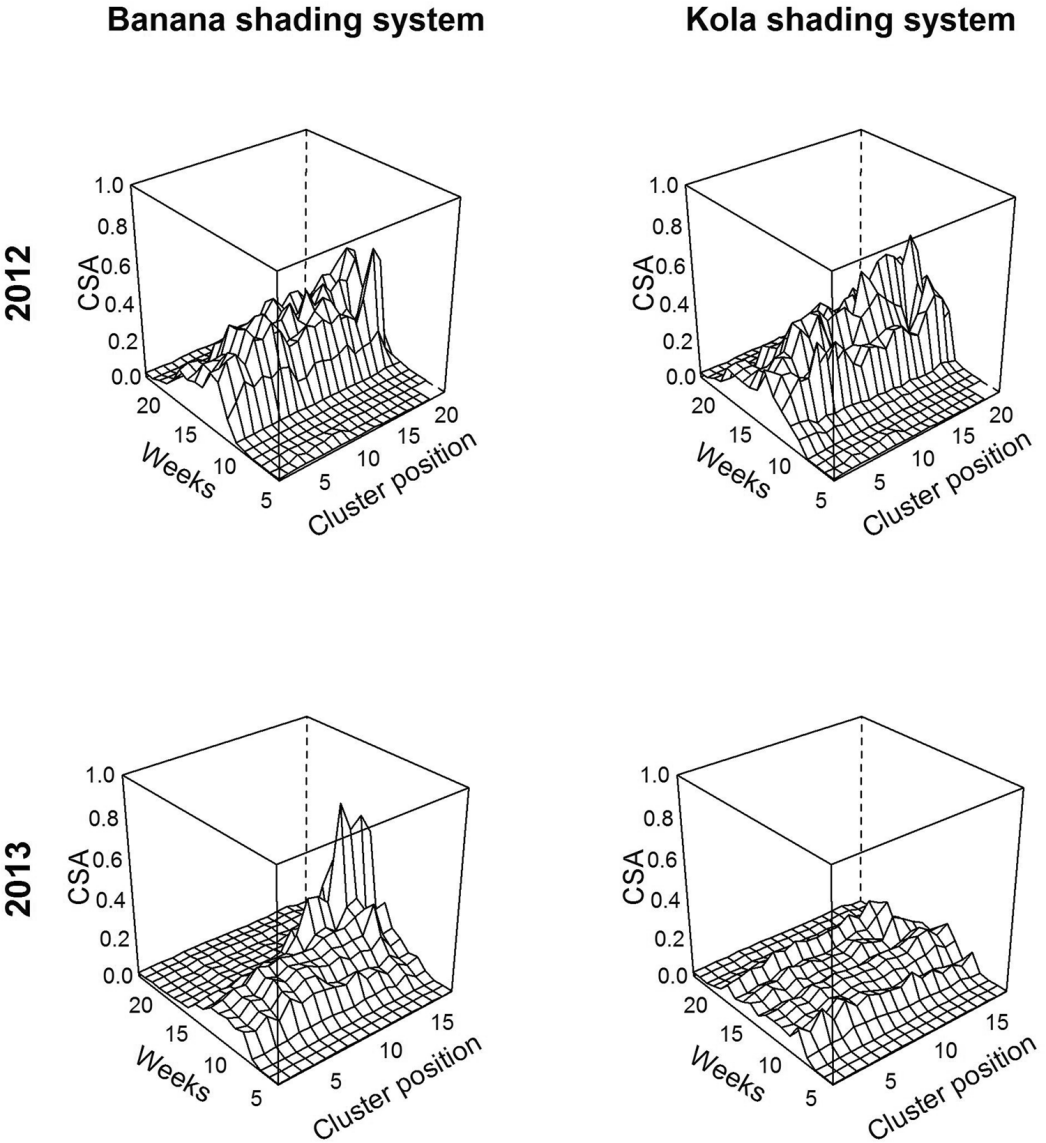
*In silico* experiment: prediction of cluster symptom appearance (CSA) dynamics along the branch in two different microclimates induced by shade. We applied the model (Annex 1) to the architectural field data, either with the microclimatic variables measured in the banana shading system (left column) or with a new set of microclimatic variables (precipitation, temperature and relative humidity) measured under the shade of a forestry tree (kola shading system) present in the coffee farm (right column), for two years, 2012 and 2013.

In the *in silico* experiments, we used the model (Annex 1) fitted with the field data (banana shading system) to predict CSA under new microclimatic conditions, namely, precipitation, temperature and relative humidity, measured under the shade of a forestry kola tree present on the farm (kola shading system) for two years, 2012 and 2013. These two years constituted two scenarios of disease dynamics in agroforestry systems relative to the field data used as the reference situation. The two scenarios led to two contrasting situations (Fig. 7). In 2012, except for disease onset, which appeared to start earlier than in the reference situation, the microclimate induced by the kola shade did not appear to affect the disease course within a tree. However, in 2013, the microclimate induced by the kola shade decreased CSA, notably at the tip of the branch, but the microclimate delayed disease extinction relative to the reference situation.

## Discussion

For the first time, to the best of our knowledge, our findings formally establish that coffee tree architecture and its interactions with microclimates drive CBD dynamics in coffee trees.

We demonstrated that the fruit load per cluster, berry physiological age and cluster position along the branch were the main architectural variables influencing CSA (Fig. 5). Notably, our results showed that during the period of disease intensification (between 10 and 20 weeks after flowering), CSA is likely to increase along the branch moving away from the trunk to the tip of the branch (Figs 4 and 6). The relation between berry position along the branch and the infection of adjacent clusters has been previously observed and suggested to be of significance by Nutman and Roberts^21^; however, to our knowledge, the significance of the relation has never been statistically evidenced. Two epidemiological mechanisms could explain this process: (i) autoinfections within a tree, through contamination between neighbouring clusters, and (ii) late alloinfections between trees, through contamination from neighbouring trees. However, our results showed a weak contribution of cluster arrangement along the branch (obtained by elimination of clusters by manual pruning) to CSA, suggesting that contamination between neighbouring clusters was not significant. This result may indicate that the dispersal distance of conidia within a tree is lower than the internode distances encountered in the coffee trees in this study. If this hypothesis is correct, then the use of dwarf varieties of coffee trees with short internodes and strong aggregation of fruits could favour disease dispersal within a tree as suggested by Avelino^22^. Nevertheless, our results are consistent with previous work by Mouen *et al*.^4^, who showed that alloinfections through inoculum splashing from tree to tree induced higher disease incidences than did autoinfections within trees. Given the relevance of disease dispersal between trees, creating natural barriers between coffee trees could provide control of CBD spread. In particular, as smallholder farmers in Cameroon usually intercrop food-producing species with coffee trees, we recommend associating tall intercrop species with coffee trees. However, our results showed a weak influence of branch story in the tree on CSA (Fig. 5). These results suggest that the architecture of the trees used for this study, topped trees showing intermediate architecture between umbrella and pyramidal architectural types, behaved more as the umbrella types described by Mouen *et al*.^2^. It would be interesting to compare our results by modelling CSA in umbrella and pyramidal types to test this hypothesis and provide recommendations on coffee tree pruning as an agroecological strategy for controlling CBD.

Our study sheds light on the complex relationships between berry susceptibility, microclimate and eco-physiological functioning of a plant.

First, the weak contribution of cluster arrangement in changing disease levels suggested that pruning berries neither affects disease spread by modifying distances between clusters nor decreases berry susceptibility by increasing the berry maturation rate^6^. However, berry weight, an important yield-determining factor resulting from differences in berry pruning, may have increased. In fact, modifications of tree architecture alter the source-sink relationships within the plant^23^. The eco-physiological functioning of the plant leads to trade-offs between tree architecture and production, playing on the components of yield (amount of fruit, seed weight, chemical composition of seeds).

Second, an inspection of our data suggested that there is a relative decrease in berry susceptibility over time, as shown by the counterintuitive relationships between CSA and suitable conditions for infection (Annex 2): most symptoms appeared when suitable conditions for infection were at low levels (before 15 weeks after flowering), while the disease died out when suitable conditions for infection were more favourable (after 17 weeks after flowering). This scenario was also observed by Muller who assigned the phenomenon to a decrease in berry susceptibility over time^9^. While the mechanistic relationship between berry maturation stage and susceptibility has still not been formally established^9^, our data suggest that host ontogenic resistance, or age-related resistance, may hinder disease development over time.

Third, our results emphasized that the suitable conditions for infection three and four weeks before CSA (SCI W2 and SCI W3, respectively) were the main climatic variables influencing CSA (Figs 3 and 4), and the suitable conditions for infection four weeks and two weeks before CSA (SCI W3 and SCI W1, respectively) were involved in substantial interactions with berry physiological age and cluster position, respectively. These results suggest that the latent period of the pathogen in host tissues could be highly variable in the field, between two to four weeks. The interaction between suitable conditions for infection and berry physiological age suggests that the latent period increases with ontogenic resistance, and overall, these results increase the need for mechanistically linking latent individuals and berry ontogenic resistance, which was not explicitly introduced in the current model.

We believe that the complex relationships between berry susceptibility, microclimate and eco-physiological functioning of a plant could be achieved by combining functional structural plant models, such as Greenlab^23^, with a CBD epidemiological model, such as susceptible-infected-exposed-removed models (SEIR models). These models have the advantage of explicitly considering latent individuals (“exposed” compartment) and ontogenic resistance in terms of eco-physiological functioning of a plant.

Given that coffee tree architecture in interaction with microclimates strongly affects CBD epidemics, strategies for coffee crop protection may consist in manipulating these features in harmony with the natural environment of the coffee tree to design naturally induced management practices. Such strategies may be developed in the context of smallholder farms in particular, where low incomes hinder the use of chemicals to manage diseases. Agroecological strategies for controlling CBD are scarce, and agroforestry systems have been recently put forward by numerous authors as efficient ways to manage CBD^4,16–20^. In this context, we explored the effects of the shade of a forestry kola tree on CSA through shade modifications of microclimates (precipitation, temperature and relative humidity). As the range of variation in the microclimatic data obtained under the kola shading system was reasonably within the range of variation in the data obtained in the banana shading system (data not shown; dataset available in the Supplementary Information files), there is good confidence in the scenario outcomes. For the two scenarios investigated using climatic data recorded over two years under the kola shading system (Fig. 2b), the microclimates had contrasting effects on CSA. In the 2012 scenario, the microclimate under the kola shading system did not change disease levels relative to those in the reference situation of the banana shading system (Fig. 7). In the 2013 scenario, however, the microclimate under the kola shading system decreased disease levels, notably, at the tip of the branch, but the microclimate increased the epidemic duration. The important variations in the epidemic behaviours predicted by the two scenarios suggest that CBD is governed by a subtle balance between microclimate and architectural variables. This exploratory work emphasizes the need to be careful when considering management of CBD in agroforestry systems and calls for further studies to improve our knowledge of microclimates generated under varying shading systems to determine their effects on the disease course.

In terms of microclimates, a possible improvement of this study would be to use rainfall intensity (which requires specific equipment for monitoring) rather than cumulative rainfall. In fact, while Waller^3^ showed a relation between cumulative rainfall and the quantity of splashed *C. kahawae* spores, our statistical analyses showed that cumulative rainfall was not important for CSA. These results suggest that under natural conditions, the relationship between rainfall and spore splashing may not be as simple as Waller^3^ has indicated, and the kinetic energy of rain droplets would likely be more accurate to consider^24,25^. Similarly, it is important to note the need to evaluate the modification of other variables induced by shade. For example, the amount of light intercepted by the coffee tree and wind speed have been shown to be altered under shade^17^, and these variables can potentially affect the disease course through modifications of the host (fruit load due to reduced floral initiation, berry maturation and susceptibility) and modifications of pathogen dispersal, respectively. These variables could be controlled by adequate management of shade cover and the selection of shade species (canopy height, shape and size) and then used as regulation instruments to adapt recommendations, as previously conducted for coffee leaf rust^24^. We suggest that contrasting the effects of shade trees on disease dynamics needs a more in-depth evaluation to identify the possible trade-offs among the environmental variables at play.

Finally, our study highlights the substantial effect of coffee tree architecture on CBD dynamics and disentangles the differential effects of architecture and microclimate on CBD. We promote the combination of epidemiological and architectural modelling, as has already been undertaken for grapevines^26^. This time-saving strategy would facilitate the design of novel, more cost-effective and environmentally friendly management strategies at both the tree scale and plot scale.

## Methods

### Field data

#### Architecture experiment

Data were collected on a coffee farm located in Bamendjou (Alt. 1600 m), West Cameroon (5°24′0″N; 10°19′0″E) over two consecutive years (2012 and 2013) from coffee trees (*Coffea arabica*) with a mixture of varieties, Java and Jamaïque, grown in association with plantain banana trees that provide partial and non-permanent shade to coffee trees. Coffee trees were planted on average at 1200–1500 trees/ha as is often conducted in traditional Cameroonian plantings^27^. Banana trees were intercropped with the coffee trees at approximately the same density. These coffee trees were approximately 15 years old and topped, showing an intermediate architecture between pyramidal architecture (branches and stories are well differentiated from the top of a tree to its base), and umbrella architecture (branches are almost in the same stories in a tree) (Fig. 1a).

A factorial trial was carried out on 345 branches from 80 coffee trees (3 branches on 45 trees in 2012 and 6 branches on 35 trees in 2013). First, to address berries of the same age, all late berries were removed manually. To evaluate disease dispersal, three distances between berry clusters were created by manually eliminating a part of the clusters on each branch of the trees. The three different cluster arrangements were obtained by removing (i) 0 clusters (0% removal corresponding to the control trees), (ii) 1 cluster out of 4 (25% removal), and (iii) 1 cluster out of 2 (50% removal) along all the branches of the coffee trees (Fig. 3).

The total number of berries, the number of diseased berries and the number of newly diseased berries (newly diseased berries were identified by a mark on each observation date) were monitored on all the sampled branches in 2012 and 2013 at the top, middle and lower stories of each studied tree. Berries were monitored weekly from fruit onset, at 5 and 4 weeks after flowering for 2012 and 2013, respectively, to 29 weeks (4 weeks before harvest) after flowering.

Temperature and relative humidity were measured hourly from the beginning to the end of the observation period using Tinytag® (Gemini, UK) dataloggers located in two randomly chosen coffee trees; one datalogger was placed in the middle story of each coffee tree, under leaves to avoid direct sunlight. To measure the quantity of daily rainfall during the observation period, two rain gauges were positioned in the plot without surrounding vegetation to avoid any rain capture perturbation.

#### *In silico* experiments

Numerical simulations aimed at identifying prospects for how agroforestry systems can affect CBD dynamics at the tree scale. We investigated whether the microclimates induced by the shade of a forestry kola tree decreased CBD levels as cited by numerous authors^4,16–20^. Those simulations involved microclimate data collected in 2012 and 2013 at the farm, under the canopy of a kola (*Cola* sp.) tree, namely, the kola shading system. Temperature and relative humidity were measured hourly during each observation period using Tinytag® dataloggers located in the middle story of one coffee tree grown under the kola tree. This coffee tree was not part of our experimental setup but was used only to obtain microclimate data. The quantity of daily rainfall under the kola tree was estimated by positioning a rain gauge under the kola canopy without surrounding coffee trees.

### Definition and construction of the variables of interest

#### Response variable

We marked diseased berries each week to detect new symptoms on a berry cluster, referred to as CSA (Table 1). CSA was defined as a binary variable valued 1 if any new diseased berry appeared and valued 0 otherwise.

#### Explanatory variables

Years (2012, 2013) were introduced as a 2-level factor (Table 1).

#### Explanatory variables related to tree architecture

Berry physiological age: Berry physiological age was used as an indicator of berry maturation that we assumed to be linked to berry susceptibility and was obtained by computing the sum of degree days from flowering (Table 1) using a base temperature of 10 °C, below which the coffee tree does not grow^28,29^.

Cluster position: The position of the berry clusters along the branch was defined as an ordinal variable ranging from position 1 (the first cluster immediately at the trunk) to the last position at the tip of the branch (Fig. 3). The last position of a cluster was variable among branches, with the maximum found at position 21.

Cluster arrangement: Cluster arrangement was defined as a factorial variable corresponding to the rates of cluster removal (0, 25 or 50% removal) (Fig. 3). Removing a cluster was achieved by removing all the berries of the cluster.

Branch story: The branch story in the coffee tree was defined as a factorial variable describing the story of the branch in the tree, namely, top, middle and lower stories.

Cluster fruit load: Finally, the fruit load variable was defined as the number of berries per cluster.

#### Explanatory variables related to microclimate

Time windows considered: The CBD latent period (time between host infection and appearance of symptoms) can vary between 6 and 15 days under controlled laboratory conditions according to Van der Vossen and Waweru^12^ and Pinard *et al*.^13^ and between 14 and 30 days in the field according to Mulinge^8^. Thus, in this study, we considered that the latent period could vary between 6 days and 1 month. We computed the microclimate covariates in 7-day time intervals corresponding to time windows of 7 to 14 days (W1), 15 to 21 days (W2) and 22 to 29 days (W3) before the appearance of cluster symptoms.

Precipitation: Rainfall is acknowledged as a key variable related to CBD development^3,4^. In our study, precipitation was defined as the cumulative rainfall (in millimetres) within each time window, W1, W2, and W3, as previously described.

Suitable conditions for infection: Berry infection (formation of germ tubes and penetration of host tissues) was found to occur at temperatures ranging from 15 to 25 °C^11^, with an optimum temperature of 20 °C for hypocotyl inoculation tests^12^. Inhibition of mycelium growth was found to occur at 30 °C^30^. At least 5 hours of wetness close to saturation (at least 98% relative humidity) at temperatures above 15 °C is required for infection to occur^3^. Based on these findings, we computed the number of hours beyond 5 hours of suitable temperature and relative humidity within each time window, W1, W2, and W3.

### Statistical analyses

Our study focused on the link between the presence (or absence) of CSA and the explanatory variables (or predictors) defined in the previous section. All statistical analyses were carried out using R version 3.4.1^31^, namely, R packages gbm v2.1.1.^32^ and dismo v1.1.4.^33^

#### Model description

We applied a statistical method from the field of machine learning, namely, BRT^34,35^, to build a stochastic, nonlinear regression model from our dataset. This method is attracting increasing interest in ecology and epidemiology^36–40^. BRT combines the advantages of regression trees (capable of handling qualitative and quantitative predictors and accommodating missing data, and prior information or data transformation is not needed) and boosting (improves prediction accuracy by combining many simple models)^36^. Regression trees find the best binary split of the data, recursively partitioning the training data set one predictor at a time. The best split is the one that maximizes the homogeneity of the response variable within partitioned groups. The boosting algorithm proceeds by iteratively fitting a new regression tree to the prediction residuals of the preceding tree; thus, the final model is an additive expansion of the regression trees. To avoid overfitting, shrinkage and cross-validation techniques were used^41^. The optimal number of boosting trees was determined using a ten-fold cross-validation procedure, retaining the number that minimized the average holdout residual deviance^33^. In our study, the response variable was the presence or absence of CSA for each week. Accordingly, we selected the Bernoulli distribution in our BRT models. Moreover, data were divided into a training set and a test set (one branch among 6 was randomly selected per tree and per week for the test set). The test set was used to evaluate the model’s predictive performance.

The main parameters of the BRT models are tree complexity (tc), which controls the interaction depth; the learning rate (lr), which controls shrinkage^42^; and the bag fraction (bf), which introduces some randomness into the model fit^41^. We assessed the appropriate model settings by testing combinations of these three parameters chosen as follows: tc ranging between 2 and 8, lr in the range {0.01, 0.005, 0.001} and bf in the range {0.75, 1}. We selected the set of values that minimized the model predictive deviance measured on the excluded folds of a cross-validation procedure. The regression model was built using the optimal setting and provided the probability of symptom appearance inside a cluster as the output.

The last step consisted in transforming the quantitative output (probability of symptoms) into a binary variable describing the presence or absence of a disease symptom, i.e., simplifying the regression model into a binary classifier. The receiver operating characteristic (ROC) curve illustrates the performance of a binary classifier as its decision threshold is varied. The curve is obtained by plotting the true positive rate, or probability of detection, against the false positive rate, or probability of a false alarm, at various threshold settings. The area under the curve (AUC) provides a metric ranging between 0 and 1 for classifier performance: the closer the AUC of a model is to 1, the better it is. We computed the AUC using the R package pROC v1.8^43^.

#### Relative influence of variables

BRT provided a measure of the relative influence of predictors on the response variable. BRT is based on the number of times a variable is selected for splitting, weighted by the squared improvement to the model as a result of each split and averaged over all trees^34,44^. The measure is scaled such that all relative influences sum to 100; high values indicate a strong influence.

#### Partial dependence plots

To visualize the marginal effect of each covariate on the response variable, a partial dependence function was calculated, averaging all the effects of all other covariates. In BRT, an approximation is calculated. Along the range of each predictor, the average of the model predictions is computed. Partial dependence plots are extremely useful for knowledge discovery in data, especially when low-order interactions and main effects predominate.

#### Relative strength of interaction effects

The strength of interaction between two predictors was evaluated using the magnitude of departure from a purely additive model. Using the R package gbm, we computed the Friedman’s H-statistic^45^ to determine the relative strength of the interaction effects. H is in the interval [0, 1], with high values indicating large interaction effects.

### *In silico* experiments

For *in silico* experiments, we applied a new set of microclimatic variables (suitable conditions for infection and precipitation) measured under a kola shade tree (the kola shading system) in years 2012 and 2013 to the fitted model (Annex 1) as described in the section “Field data”.

## Supplementary information

Supplementary information

Dataset 1

## Data Availability

The datasets generated or analysed during this study are included in this article (and its Supplementary Information files).
