# Supplementary material for: Coffee tree architecture and its interactions with microclimates drive the dynamics of coffee berry disease in coffee trees
Source: Sci Rep. 2019 Feb 22;9:2544. doi: 10.1038/s41598-019-38775-5 (PMC6385237; doi:10.1038/s41598-019-38775-5)

**Supplementary information**

**Coffee tree architecture and its interactions with microclimates drive the dynamics of coffee berry disease in coffee trees**

Natacha Motisi*, Fabienne Ribeyre & Sylvain Poggi

**Annex 1. Model evaluation**

We fitted a stochastic, nonlinear regression model to the response variable presence or absence of CSA for each week.

The regression model showed satisfactory performances in terms of goodness-of-fit and generalization capacity. Correlation between predictions and observations from the training set was good (54%) and remained satisfactory in the cross-validation (43%). Simplified as a binary classifier, the model yielded a value of 0.89 for the area under the ROC curve (ROC AUC) at the optimal threshold 0.09, indicating good predictive performance. The sensitivity and specificity of the model were 0.83 (with a 95% confidence interval [0.81, 0.85]) and 0.80 (with a 95% confidence interval [0.79-0.81]), respectively. When applied to the test set, composed of 4593 cases split in 4226 absences and 367 presences of CSA, the classifier yielded 824 false positives and 71 false negatives. Overall, the classification error equaled 19.5%, which we considered as satisfactory for our purpose.

The set of parameters providing the best predictive model performance was defined by the number of trees N=2400, the shrinkage parameter lr=0.01, the tree complexity tc=8 and the bag fraction bf=0.75.

**Annex 2. Counterintuitive relationships between cluster symptom appearance (CSA) and suitable conditions for infection (SCI) over time.**

Most of the cluster symptom appearance (CSA, %) occurred when suitable conditions for infection (SCI W3 was used for the demonstration) were at low levels (before 15 weeks after flowering), while disease died out when suitable conditions for infection were more favorable (after 17 weeks after flowering).

Dots represent CSA (red dots) and SCI W3 (blue dots) averaged by cluster arrangement modality (Table 1, Fig. 1) and year (2012, 2013). Lines represent the mean data obtained with a 3-degree polynomial model.


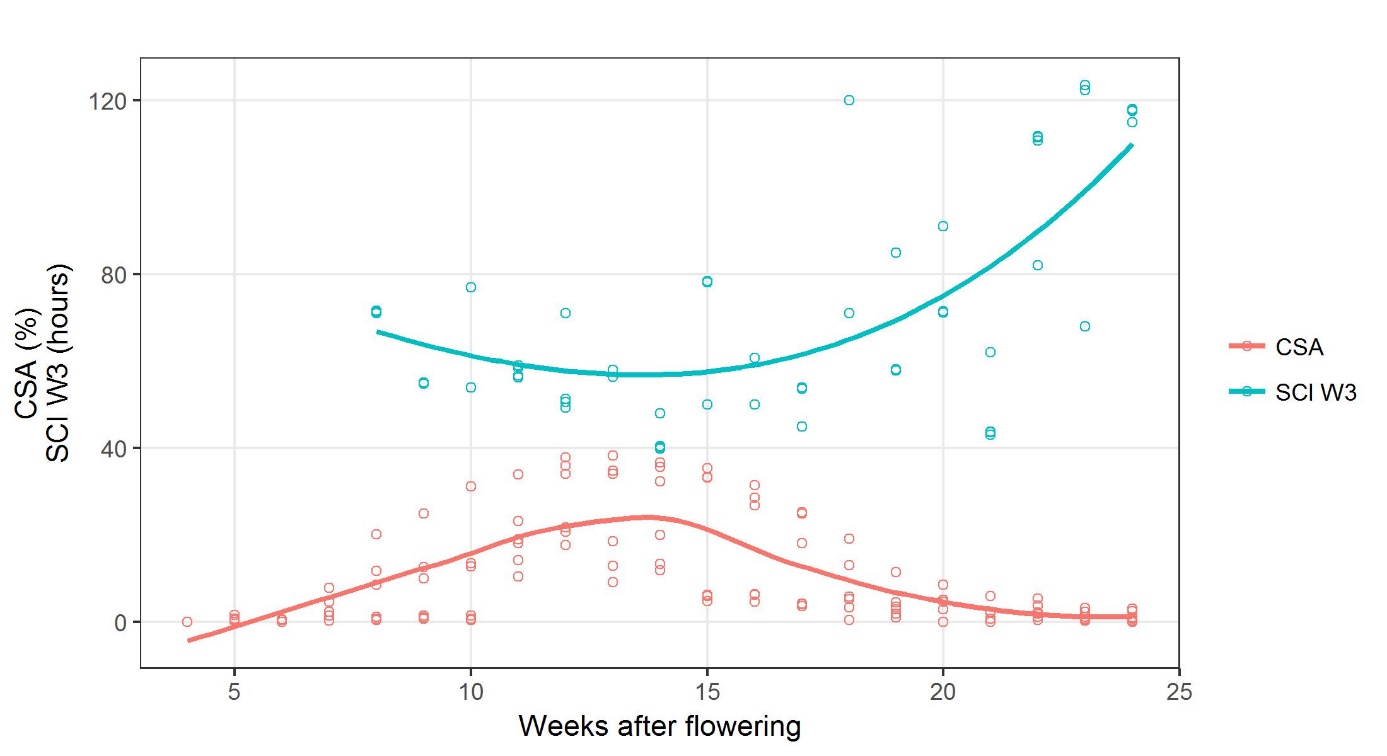

Supplement: Supplementary file 1 — Supplementary information [file 41598_2019_38775_MOESM1_ESM.docx]
